# MG2C: a user-friendly online tool for drawing genetic maps

**DOI:** 10.1186/s43897-021-00020-x

**Published:** 2021-12-09

**Authors:** Jiangtao Chao, Zhiyuan Li, Yuhe Sun, Oluwaseun Olayemi Aluko, Xinru Wu, Qian Wang, Guanshan Liu

**Affiliations:** 1 Key Laboratory for Tobacco Gene Resources, Tobacco Research Institute of Chinese Academy of Agricultural Sciences, Qingdao, 266101 China; 2 Graduate School of Chinese Academy of Agricultural Sciences, Beijing, 100081 China

**Keywords:** Genetic map, Online tool, Perl, MG2C

## Abstract

Genetic map is a linear arrangement of the relative positions of sites in the chromosome or genome based on the recombination frequency between genetic markers. It is the important basis for genetic analysis. Several kinds of software have been designed for genetic mapping, but all these tools require users to write or edit code, making it time-costing and difficult for researchers without programming skills to handle with. Here, MG2C, a new online tool was designed, based on PERL and SVG languages.

Users can get a standard genetic map, only by providing the location of genes (or quantitative trait loci) and the length of the chromosome, without writing additional code. The operation interface of MG2C contains three sections: data input, data output and parameters. There are 33 attribute parameters in MG2C, which are further divided into 8 modules. Values of the parameters can be changed according to the users’ requirements. The information submitted by users will be transformed into the genetic map in SVG file, which can be further modified by other image processing tools.

MG2C is a user-friendly and time-saving online tool for drawing genetic maps, especially for those without programming skills. The tool has been running smoothly since 2015, and updated to version 2.1. It significantly lowers the technical barriers for the users, and provides great convenience for the researchers.

## Introduction

Genetic map, also known as linkage map, illustrates the arrangement and physical or genetic distance between molecular markers (genes or DNA markers) on chromosomes (Saraswathy and Ramalingam 2011). It plays a vital role in the field of life science, including genetic breeding, disease detection, gene map cloning and other scientific researches. Genetic maps can provide researchers with more reliable information and great convenience. With the rapid development of sequencing technology, genomic information of more species has been deciphered, and extensive molecular markers have been identified and utilized (vanDijk et al. 2018). And high-quality genetic maps turn to be an important basis for genetic analysis. Thus, it is necessary to develop different visualization tools to meet the needs of those researchers with different knowledge foundations.

At present, there are several tools developed to draw genetic maps, including MapChart (Voorrips 2002), LinkageMapView (Ouellette et al. 2018), MapDraw (Liu and Meng 2003), etc. MapChart is a classic genetic map viewer tool, available for the MS-Windows only. It requires users to first master the command syntax and then write the command line to complete the genetic map drawing (Voorrips 2002). LinkageMapView is a free extension package of R language which requires an R language environment, and is mainly used for QTL genetic map (Ouellette et al. 2018). The first step to use LinkageMapView is setting up R language environment, then learning relevant command syntax, and the finally step is writing code. MapDraw is a simple viewer tool of genetic map, and it is constructed based on EXCEL macro code (Liu and Meng 2003). To get a map, users are required to modify the existing code template. These tools are now widely used for the visualization of genetic maps in various research fields, however, the requirement that the users need to master certain programming ability seriously hinders the progress of numerous research work, and it also seriously limits the number of users.

In view of this, MG2C, an online visualization tool for genetic map was developed, based on PERL and SVG languages. The tool can be used on any operating system, and the latest version is MG2C2.1. After preparing the information including gene location and chromosome length, according to the EXCEL template, the users only need to open an internet browser that supports SVG such as Google Chrome or FireFox that support SVG display, enter the URL (http://mg2c.iask.in/mg2c_v2.1), and submit the corresponding information into the input section in operation interface, a standard genetic map will be generated. The map can be downloaded and saved in SVG format for further editing and beautification. In this report, the workflow, main parameters and usage of MG2C are mainly introduced.

## Results

### Workflow

**Fig. 1:**
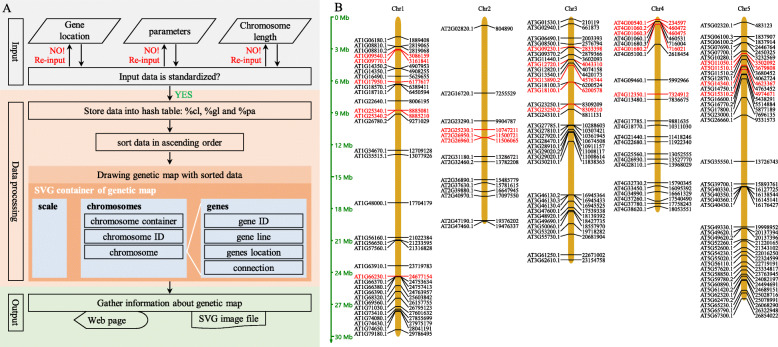
**A** Workflow of MG2C. **B** The genetic map of *MYB* gene family from *A*. *thaliana* generated by MG2C

The workflow of MG2C consists of three steps: data input, data processing and output (Fig. 1A). (1) The input data include: gene location, parameters and chromosome length. Generally, users can draw a genetic map with default parameters. After submitting the input data, MG2Cconfirms whether the input data are standard before proceeding to the next step. (2) During data processing, the input data initially stored into the hash table (Fig. 1A) first are sorted in ascending order based on the gene location, and then visualization process initiates. The drawing of a genetic map is decomposed into 8 modules inMG2C program as follows: SVG container, single chromosome container, chromosome, chromosome ID, gene lines, gene ID, connection and scale. (3)The visualization results are finally stored into an image file and displayed on the webpage. Besides SVG format, MG2C also provides jpeg, png or tiff format option.

### Main parameters

In this paper, all the parameters are divided into 8 modules: SVG container, single chromosome (chr) container, chromosome, gene line, gene ID, connection and scale. Each module corresponds to its own attribute parameters (Table 1) with a total of 33 parameters.

**Table 1:** Default values and description of MG2C main parameters

Modules	Parameters	Default values	Description
SVG container	Width	1000 px	Width of SVG container
	Height	900 px	Height of SVG container
Single chromosome (chr) container	Width	270 px	Width of single chr container
	Height	400 px	Height of single chr container
	Fill	None	Fill color of single chr container
	Border-width	1 px	Border width of single chr container
	Border-color	None	Border color of single chr container
Chromosome ID	Font	Times New Roman	Font family of chr ID
	Size	12 px	Font size of chr ID
	Color	Black	Font color of chr ID
Chromosome	Width	10 px	Width of single chr
	Height	300 px	Height of single chr
	Fill	None	Fill color of single chr
	RX	14 px	Rectangular round angle X of single chr
	RY	14 px	Rectangular round angle Y of single chr
	Border-width	1 px	Border width of single chr
	Border-color	Black	Border color of single chr
Gene lines	Color	Black	Gene lines’ color
	Width	0.5 px	Gene lines’ width
	Type	1	Gene lines’ type
Gene ID	Font	Times New Roman	Font family of gene ID
	Size	11 px	Font size of gene ID
	Color	Black	Font color of gene ID
	Margin	15 px	Margins between gene ID and Chr
	Display_type	1	Display types of gene ID
Connection between gene ID and gene line	Color	Black	Line color of connection
	Width	0.5 px	Line width of connection
Scale	Units	bp	Units of scale, base pair (bp) or centimorgan (cM).
	Width	20 px	Width of genetic map scale
	Position_X	20 px	Position X of genetic map scale
	N_tick mark	10	N mark lines of each unit
	Decimal place	0	Significant digit
	Color	Black	Color of scale

### Usage

#### Data input

Users are required to prepare the input data for MG2C, according to an excel template provided on the website. The input data are divided into two parts. One is gene location (input1), and the other is chromosome length (input2). The former contains 5 fields: gene_ID, gene_start, gene_end, chr_ID and gene_color. Gene_color is an optional field used to customize color of the gene ID, with the default in black. The latter contains two fields: chr_ID and chr_length. The delimiter between the fields is “TAB”. Users copy and paste the input data into the corresponding textbox, left-click the “DRAW” button and a genetic map will be generated by MG2C. In addition, frequently asked questions (FAQ) in English and Chinese version separately are provided on the website.

Examples

Here, chromosome information of 163 *MYB* gene family members in *Arabidopsis thaliana* was obtained (Dubos et al. 2020). A genetic map was generated by MG2C (Fig. 1B), showing the overall distribution of *MYB* gene family on chromosomes and the corresponding position of 27 important gene members.

## Discussion

MG2C is a simple and user-friendly online tool for visualizing genetic maps. To draw a genetic map, users only copy-paste the data of gene location and chromosome length into input1 and input2 textbox of MG2C, respectively; and left-click the “DRAW” button to get a standard genetic map quickly. The output result can also be saved as a SVG file for further re-editing. In addition, users can change the value of all parameters of MG2C based on their preference. Briefly, MG2C is well-designed software, avoiding users writing or editing code. It not only saves the analysis time of users but also meets the needs of those without adequate programming skills. Since 2015, the tool has been running smoothly for about 5 years, and will be updated with a constant functional improvement and free service in future. By the end of March 2021, the number of unique access IP was over 28,000.

## Data Availability

The URL of MG2C is http://mg2c.iask.in/mg2c_v2.1/. The source code of MG2C has been deposited into github (https://github.com/chaojiangtao985/MG2C/tree/mg2c_web_v2.1).
